# Behind the Wheel of a Truck Simulator: Comparison of Self-Reported, Performance-Based, and Simulation Methods for Predicting Driver Traffic Offences

**DOI:** 10.3390/bs16020271

**Published:** 2026-02-12

**Authors:** Paulina Baran, Piotr Zieliński, Mariusz Krej, Marcin Piotrowski, Łukasz Dziuda

**Affiliations:** 1 Department of Psychophysiological Measurements and Human Factor Research, Military Institute of Aviation Medicine, 01-755 Warsaw, Poland; mkrej@wiml.waw.pl (M.K.); ldziuda@wiml.waw.pl (Ł.D.); 2 Department of Aviation Psychology, Military Institute of Aviation Medicine, 01-755 Warsaw, Poland; pzielinski@wiml.waw.pl; 3 Department of Simulator Studies and Aeromedical Training, Military Institute of Aviation Medicine, 01-755 Warsaw, Poland; mpiotrowski@wiml.waw.pl

**Keywords:** traffic offences, assessment methods, truck drivers, car simulators, road traffic safety

## Abstract

Traffic violations represent a significant public health concern, with professional drivers substantially impacting road safety. This pilot study compared self-report questionnaires (general personality versus domain-specific), performance-based tests, and driving simulator measures to determine which assessment method best predicts traffic offences among professional truck drivers. Participants (*N* = 27) completed the Impulsiveness–Venturesomeness–Empathy Questionnaire (IVE), the Road Traffic Behaviours Questionnaire (KZD), and the Vienna Risk-Taking Test Traffic (WRBTV) and performed standardised driving scenarios in a truck simulator. Performance was assessed using speed variations in five validated decision-making situations. Drivers were classified into two groups based on relatively higher and relatively lower numbers of self-reported traffic offences. The KZD demonstrated the strongest group differentiation (*p* = 0.034, *d* = 0.76). Simulator performance was significantly different between the groups (*p* = 0.033, *d* = −0.68), with offence-reporting drivers showing smaller speed reductions. The WRBTV and the IVE empathy subscale approached significance (*p* = 0.056 and *p* = 0.059, respectively). Higher empathy characterised offence-free drivers, suggesting social–emotional factors may contribute to traffic safety. General impulsiveness and venturesomeness showed no group differences. The results indicate that domain-specific questionnaires and behavioural assessments offer superior predictive validity compared to general personality measures for identifying potentially unsafe drivers. ROC analysis revealed moderate predictive validity across significant measures (AUC: 0.64–0.70), with differential patterns of sensitivity and specificity among predictors. The findings suggest implementing tiered screening approaches using domain-specific questionnaires as initial cost-effective tools, followed by simulator assessment for at-risk drivers, enabling transport companies and regulatory bodies to identify high-risk drivers proactively.

## 1. Introduction

Road traffic safety represents a significant global public health challenge, with professional drivers playing a crucial role in this complex system. According to the World Health Organization (2018), road traffic crashes cause 1.35 million deaths annually and are the leading killer of people aged 5–29 years worldwide. Recent data from Poland highlight the disproportionate impact of heavy goods vehicles on road safety. Although heavy vehicle crashes represent only about 3% of all Polish road accidents, they result in significantly higher fatality rates, i.e., 19 fatalities per 100 crashes compared to an average of 11 for all vehicles (BRD24.PL, 2024). Therefore, understanding the factors behind these concerning statistics is essential for developing effective countermeasures.

Meanwhile, road safety research consistently identifies human behaviour as the dominant factor in vehicle accidents (e.g., Brown et al., 2017; Bucsuházy et al., 2020; Chang et al., 2019). A comprehensive review by Zainuddin et al. (2023) quantified this relationship in the context of heavy goods vehicles, revealing that human factors account for 50% of studied accident causes, significantly outweighing both environmental (26.5%) and vehicle-related factors (23.5%). These human factors encompass a range of problematic behaviours, including distraction, fatigue, regulatory non-compliance, and perhaps most critically, speeding. Indeed, the prevalence of speed-related violations among commercial drivers is particularly concerning. Tseng et al. (2016) documented that over 11% of large-truck operators reported receiving at least one speeding citation within a single year. Such traffic violations represent more than mere regulatory infractions, i.e., they constitute a significant public health concern, as risky driving behaviours directly contribute to road casualties and their associated societal costs (see Amoadu et al., 2023). For this reason, the relationship between driver behaviour and accident risk creates an urgent imperative for effective assessment and intervention strategies. And with commercial vehicles logging millions of kilometres annually on public roadways, the potential safety benefits of identifying at-risk drivers before incidents occur are substantial. Therefore, applying robust driver assessment methods could significantly impact overall traffic safety and reduce the considerable public health burden associated with commercial vehicle accidents.

To address this need, current approaches to driver evaluation encompass diverse methodologies, each with distinct theoretical foundations and practical implications for road safety. Primarily, traditional personality assessments, such as the IVE (Impulsiveness, Venturesomeness, Empathy) questionnaire (Eysenck & Eysenck, 1978), represent one methodological approach that measures broad personality dimensions hypothesised to influence risk-taking. Meta-analytic evidence by Akbari et al. (2019), analysing over 11,000 participants, demonstrated associations between risky driving and traits like low agreeableness and high sensation-seeking. However, subsequent research has revealed significant limitations in applying these general measures to specific populations. Studies by Lucidi et al. (2019) and Brown et al. (2017) demonstrated that personality–behaviour relationships vary significantly across age groups and driver categories. Notably, while Baran et al. (2021) found that impulsiveness significantly predicted risky driving in general driver populations—even regardless of risk perception—such robust associations may not generalise uniformly to professional drivers operating under distinct occupational constraints and selection pressures. Therefore, such findings highlight the fundamental limitation of using stable personality traits to predict dynamic, context-dependent driving behaviours, particularly among truck drivers who work in unique professional conditions.

Given these limitations of general personality measures, researchers have developed domain-specific questionnaires that directly assess attitudes and self-reported behaviours in traffic contexts. This methodological shift recognises that instruments closely aligned with the criterion behaviour may yield stronger predictive validity than broad personality assessments. Accordingly, a wide variety of specialised driving behaviour questionnaires have been developed to address specific driver populations and contexts. For general driver assessment, instruments such as the KZD (Road Traffic Behaviours Questionnaire; Baran et al., 2021) measure tendencies toward risky behaviours in traffic situations. The KZD was specifically designed to assess the frequency of engaging in risky driving behaviours identified in the literature as common causes of road accidents, focusing on actual traffic behaviours rather than general personality traits (Baran et al., 2021). Unlike the widely used Driver Behaviour Questionnaire (DBQ), which categorises violations, errors, and lapses based on Reason’s error taxonomy (Reason et al., 1990), the KZD employs a frequency-based assessment of specific risky behaviours on a 4-point Likert scale (0 = never to 3 = often), making it particularly suitable for identifying drivers with elevated propensity for risk-taking in traffic situations (Baran et al., 2024; Eysenck & Eysenck, 1978). This domain-specific focus aligns with the principle of criterion–predictor correspondence, whereby measures closely matched to the target behaviour demonstrate superior predictive validity (Baran et al., 2021; Taubman-Ben-Ari et al., 2016). Other approaches include multilevel assessment of unsafe actions (Jomnonkwao et al., 2022) and evaluation of peer pressure influence on young drivers’ risk behaviours (Li et al., 2025). For professional drivers specifically, researchers have developed instruments that account for their unique working conditions and typical behavioural repertoires. Useche et al. (2021) validated the F-DBQ (Freight Driving Behaviour Questionnaire), a concise tool adapted for long-haul professional drivers. Similarly, other researchers have created specialised versions of the Driver Behaviour Questionnaire (DBQ) for various professional contexts, including a 15-item version for public transport drivers (Caird & Kline, 2004), a 21-item adaptation for Bus Rapid Transit operators (Useche et al., 2017), a 25-item version comparing professional and non-professional drivers (Maslać et al., 2018), and a 20-item version tested on drivers from a community-based nursing organisation (Newnam & VonSchuckmann, 2012). Noteworthy, these specialised instruments recognise that different segments of the driving population face distinct task-related conditions that influence their risk behaviours.

However, when evaluating domain-specific driving questionnaires, researchers must also acknowledge several significant methodological limitations inherent to self-report measures. Primarily, while these instruments benefit from content validity, they fundamentally rely on accurate self-perception and honest reporting, i.e., qualities that may be particularly compromised in high-stakes contexts such as driver rehabilitation programmes, occupational screening, or post-accident evaluations, where respondents have clear incentives to present themselves favourably. Moreover, a particularly significant concern is that meta-analytic evidence suggests that self-reported traffic data may produce inflated effect sizes compared to archival records, potentially due to common method variance effects (Barraclough et al., 2016). Recent research by Odachowska-Rogalska et al. (2024) provides further evidence of self-report limitations, documenting a significant “awareness gap” wherein drivers readily identify risky and aggressive behaviours in other road users while failing to acknowledge similar behaviours in themselves. Alonso et al. (2022) corroborate these findings, demonstrating that drivers consistently rate their own behaviours more favourably than those of other drivers, while pedestrians assess driver behaviours significantly more negatively than drivers themselves—a perceptual discrepancy that highlights the subjective nature of self-reported driving data.

On the other hand, despite these methodological challenges, empirical evidence supports the value of well-designed self-report instruments in transportation safety research. Taubman-Ben-Ari et al. (2016) provide empirical support for their validity in predicting actual driving behaviours, finding significant correlations between self-reported driving styles and objective measures collected via both in-vehicle data recorders and driving simulators. Recent research has further demonstrated that driver subgroups identified using domain-specific instruments can significantly differ in registered traffic offences, supporting their value for identifying high-risk drivers (Martinussen et al., 2017). Notably, drivers characterised by intentional violations rather than unintentional errors showed increased likelihood of official traffic offences, suggesting these instruments can meaningfully differentiate between types of problematic driving behaviour. Collectively, these findings indicate that carefully designed self-report instruments can serve as reliable indicators of real-world driving behaviours, particularly when measuring habitual driving patterns and tendencies, and especially when combined with complementary assessment methods.

While self-report tools, whether general or domain-specific, offer practical advantages for large-scale assessment, the search for more objective measurement approaches has led to the development of performance-based methods, which offer more direct measurement of driving tendencies with reduced susceptibility to reporting biases. Performance-based measures, like, for example, the Vienna Risk-Taking Test—Traffic (WRBTV; Hergovich et al., 2011), attempt to capture actual behavioural tendencies through standardised tasks. The WRBTV is a computerised test based on Wilde’s theory of risk homeostasis that presents 24 traffic situations (following one practice item), each shown twice to participants. During the first viewing, participants observe the situation; during the second viewing, they indicate the critical distance from a potential hazard at which a described driving manoeuvre becomes too dangerous to perform (Hergovich et al., 2011). Unlike pure self-report measures, this performance-based format requires concrete behavioural decisions in response to visual traffic scenarios, potentially reducing social desirability bias while yielding a quantifiable measure of willingness to accept risk in traffic situations (Cronbach’s *α* = 0.92) (Hergovich et al., 2011). Notably, research specifically examining WRBTV’s validity for predicting traffic offences among professional drivers remains limited in the current literature. Overall, regardless of the above, while these methods reduce self-report bias, they may lack ecological validity when administered in laboratory settings divorced from the complex traffic environment where safety behaviours actually occur. Simultaneously, driving simulators represent a more sophisticated performance-based approach, offering an optimal compromise between ecological validity and standardisation by allowing observation of behaviour in controlled yet realistic scenarios.

The utility of driving simulators has been particularly well-documented for both research and practical applications in professional driver assessment. Research by Ka et al. (2020) successfully implemented surrogate safety measures in driving simulators to evaluate crash potential and risky driving patterns among commercial drivers, demonstrating that simulator-based protocols can effectively differentiate risk levels in this population. Similarly, Mullen et al. (2011) confirmed in their comprehensive review that high-fidelity simulators provide valid assessments of performance measures such as speed control and risky traffic behaviours—metrics particularly relevant for professional drivers whose safety performance has substantial public health implications. Moreover, Pešić et al. (2022) demonstrated that simulator-derived behavioural measures can function as predictors of real-world outcomes, successfully employing logistic regression modelling with simulator data to predict driver compliance with speed limits.

Noteworthy, while individual assessment methods have been extensively validated, comparative research directly examining their relative effectiveness for predicting traffic offences remains surprisingly scarce, particularly in professional driver populations. This gap is especially evident when considering studies that jointly employ multiple methods, i.e., self-report questionnaires, performance-based tests, and simulation, to compare their predictive validity against the same criterion. Most existing research employs single-method designs that preclude such direct comparisons. For instance, Chen et al. (2025) recently found significant associations between risky driving behaviours as measured by domain-specific questionnaires and both personality traits and traffic accidents in Chinese drivers aged 55–65 years, yet their study employed only self-report methodology. Similarly, most simulator validation studies (Ka et al., 2020; Michaels et al., 2017; Pešić et al., 2022) focus on demonstrating ecological validity rather than comparing simulator metrics against other established assessment approaches. This methodological isolation limits our understanding of which assessment tools provide optimal predictive validity when resources must be allocated strategically for driver screening programmes.

The Polish context adds particular urgency to this research question. Polish professional drivers operate under unique regulatory and infrastructural conditions that may differentially affect the validity of various assessment methods. Yet, to our knowledge, no published research has systematically compared self-report, performance-based, and simulation methods within this specific population and national context. This gap limits the evidence base available to Polish transportation authorities and companies seeking to implement effective, evidence-informed driver screening protocols.

In summary, while personality questionnaires, domain-specific self-report measures, and simulator-based assessments each measure different aspects of driver characteristics and behaviours as outlined above, the relative effectiveness of these approaches in predicting traffic offences remains unclear without direct comparison. Specifically, three critical questions remain unanswered: (1) Do behavioural methods (performance-based tests and simulators) demonstrate superior predictive validity compared to self-report measures when assessed against the same criterion? (2) Among self-report instruments, do domain-specific driving questionnaires outperform general personality assessments in identifying at-risk professional drivers? (3) Can these methods be combined to enhance predictive accuracy beyond what any single approach achieves? Therefore, this research gap suggests an opportunity for developing more evidence-informed screening approaches that might contribute to road safety initiatives. The need for such comparative research seems particularly relevant for professional driver populations, who may have a significant influence on traffic safety due to their vehicle size, extensive road exposure, and potential interaction with other vulnerable road users.

Considering the above, the present pilot study addresses this evolving research area by directly comparing self-report, performance-based, and simulation methods in predicting documented traffic offences among professional drivers. Hence, the main objective is to determine which assessment method, i.e., self-report questionnaires (general personality vs. domain-specific), performance-based tests, or driving simulator measures, demonstrates the highest predictive validity for real-world traffic violations committed by truck drivers.

This study is grounded in several complementary theoretical frameworks. First, Wilde’s risk homeostasis theory (Wilde, 1982, 1994) provides the foundation for understanding how drivers maintain subjectively acceptable levels of risk through behavioural adjustments in response to perceived danger. Second, the principle of criterion–predictor correspondence (Fishbein & Ajzen, 1975) suggests that assessment tools closely matched to target behaviours should demonstrate superior predictive validity compared to general measures. Third, ecological validity theory (Brunswik, 1956) posits that assessments conducted in realistic contexts should better capture real-world behaviour than abstract measures. These frameworks collectively guided our methodological approach and hypothesis formulation.

For clarity, we define the key constructs in this study as follows: ‘Traffic offences’ refer to documented violations of traffic regulations as self-reported by participants; ‘Driving performance’ refers to observable driving behaviours measured through simulator metrics, specifically speed modulation patterns in response to potential hazards; ‘Risk-taking behaviour’ is operationalised as the willingness to accept shorter safety margins in potentially hazardous situations, and ‘predictive validity’ refers to the ability of assessment methods to differentiate between drivers with and without documented traffic offence histories.

All in all, based on the theoretical and empirical literature reviewed above, we formulated the following specific hypotheses:

**H1:**  *Domain-specific self-report measures (KZD) will demonstrate superior predictive validity compared to general personality traits (IVE scales) in differentiating drivers with and without traffic offence histories, consistent with the principle of criterion–predictor correspondence (Baran et al., 2021; Martinussen et al., 2017; Taubman-Ben-Ari et al., 2016).*

**H2:**  *Performance-based behavioural methods (WRBTV and simulator metrics) will show stronger discriminative power than self-report measures, given their reduced susceptibility to social desirability bias and enhanced ecological validity (Ka et al., 2020; Michaels et al., 2017; Pešić et al., 2022).*

**H3:**  *Among simulator-derived metrics, speed variability measures will significantly differentiate between driver groups, reflecting difficulties in maintaining consistent speed control across varying demands—a pattern previously associated with accident involvement (Af Wåhlberg, 2008; Baran et al., 2026, manuscript submitted for publication).*

**H4:**  *General personality traits related to impulse control (impulsiveness, venturesomeness) will show weak associations with traffic offences in this professional driver sample. While such traits demonstrate robust predictive validity in general driver populations (Baran et al., 2021), we predict that occupational selection effects and extensive driving experience may attenuate these relationships in professional drivers (Af Wåhlberg et al., 2017; Tseng et al., 2016).*

In addition, the specific aims include

- Comparing the discriminative power of each method in differentiating drivers with and without traffic offence history;
- Examining whether behavioural methods outperform self-report measures and assessing the feasibility of multi-method assessment protocols;
- Exploring the relative contribution of general personality traits versus driving-specific assessments to the identification of drivers who may pose elevated risks to road safety and hence public health.

## 2. Materials and Methods

### 2.1. Study Design and Experimental Setup

This pilot study employed a between-subjects design for analytical comparison. Participants were classified into two groups based on cluster analysis: those with relatively higher numbers of self-reported traffic offences and those with relatively lower numbers (see Section 2.3 for detailed methodology). The predictor variables (independent variables) were (1) general personality traits measured by the IVE Questionnaire (Impulsiveness, Venturesomeness, and Empathy subscales), (2) domain-specific risky driving behaviours measured by the Road Traffic Behaviours Questionnaire (KZD), (3) performance-based risk assessment measured by the Vienna Risk-Taking Test Traffic (WRBTV), and (4) simulator-based driving performance measured by speed variability metrics. The criterion variable (dependent variable) was group membership based on traffic offence history.

Moreover, this pilot study employed a within-subjects repeated-measures design to compare the discriminative validity of three assessment approaches: self-report questionnaires, performance-based tests, and driving simulator measures. We hypothesised that domain-specific and behavioural measures (KZD, WRBTV, simulator) would demonstrate stronger discriminative power than general personality measures (IVE) in differentiating these groups. All participants completed the full battery of assessments in a single session lasting approximately one hour. The study was conducted at the Military Institute of Aviation Medicine (WIML) in Warsaw, Poland, using a high-fidelity truck simulator with a six-degree-of-freedom motion system. Testing occurred during non-driving hours when participants were rested and healthy, ensuring optimal conditions for performance assessment.

#### 2.1.1. Participants and Procedure

The research involved 27 male professional truck drivers aged 30–62 years (*M* = 41.59, *SD* = 7.74) who successfully completed all study procedures. Three additional participants began the study but withdrew due to simulator sickness symptoms, a common occurrence in simulator research. However, with regard to the repeated measurement research design, where the assumed statistical significance is 0.05 and the statistical test power is 0.8, the minimum group size required to detect a moderate effect (*d* = 0.5) is 27 people. Additionally, previous experience with simulator studies also indicates that this number is sufficient for drawing conclusions in preliminary studies (Dziuda et al., 2014).

All participants were actively employed as professional truck drivers at the time of data collection, with daily driving as part of their occupational responsibilities and as an inclusion criterion for the study. They held valid Category C driving licences required for commercial truck operation in Poland. Their professional driving experience ranged from 2 to 35 years (*M* = 13.93, *SD* = 8.03), representing both relatively novice and highly experienced professional drivers. None of the participants had previous exposure to this particular truck simulator used in the study, though some had general familiarity with driving simulation technology through professional training contexts. Participants were recruited through a referral-based sampling method. Initial telephone contact was made with professional truck drivers who had previously participated in our research studies. These drivers provided contact information for colleagues from various transportation companies operating in Poland. Participation was entirely voluntary, and all participants received standardised financial compensation (a fixed amount predetermined in the project budget and equivalent for all participants). Data collection took place between January and February 2024.

Following ethical approval and written informed consent, participants completed assessments individually. To minimise learning effects, all participants completed a standardised familiarisation drive before the experimental scenarios, allowing them to adapt to the simulator’s controls and dynamics. The protocol began with this familiarisation phase, followed by the main driving scenarios. The order of experimental and control scenarios was counterbalanced across participants to control for potential order effects. Participants then completed the WRBTV and questionnaires in a fixed order. The entire session was designed to be completed within approximately one hour to minimise fatigue effects. Testing occurred during non-driving hours when participants were rested and healthy. Any participant reporting fatigue symptoms was offered breaks or rescheduling opportunities.

#### 2.1.2. Ethics

This research adhered to ethical principles outlined in the Declaration of Helsinki. Participants’ rights to confidentiality and data protection were prioritised throughout the study process. Professional drivers were individually approached and invited to participate, with emphasis on the voluntary nature of their involvement. Prior to any data collection, each participant received detailed explanations regarding the scientific purpose and methodological approach of the investigation. Written informed consent was obtained from all drivers before their participation in simulator sessions and questionnaire completion. All procedures followed a predetermined research protocol that received approval from the appropriate institutional authorities. No personally identifiable information was retained in the final dataset to preserve full anonymity.

### 2.2. Measures

#### 2.2.1. Driving Simulator Assessment

The study employed a high-fidelity truck simulator housed at the Military Institute of Aviation Medicine (Warsaw, Poland). This specialised equipment features a six-degree-of-freedom motion platform that accurately reproduces the physical sensations of vehicle movement, enhancing immersion and ecological validity (Baran et al., 2019). The simulator system records multiple driving parameters at 60 Hz frequency, including vehicle position, speed, acceleration, steering angle, and pedal inputs, providing high-resolution temporal data throughout each scenario. The visual display system offers a 180-degree horizontal field of view, replicating the visual perspective from a truck cabin. Force-feedback steering and realistic pedal resistance further contribute to the simulator’s fidelity, creating an immersive driving environment that closely approximates real-world truck operation. A detailed technical description of this simulator, including photographs of the cabin and instructor station, is provided in Baran et al. (2019).

- Experimental design and scenario rationale

The simulator protocols consisted of two distinct driving scenarios: an experimental scenario designed to test risky driving tendencies and a control scenario covering the same route without risk-provoking elements. This dual-scenario approach enabled isolation of behavioural responses specifically attributable to decision-making situations rather than general driving style or route characteristics. To counterbalance potential order effects, participants were randomly assigned to begin with either scenario. Each participant received a standardised introduction to the simulator’s operation and was explicitly instructed to drive naturally, reflecting their everyday driving habits.

- Decision-making situations: Design and correspondence with assessment instruments

During the experimental drives, participants encountered 12 decision-making situations distributed along an approximately 15 min route. These situations were specially designed to present drivers with choices between safer and riskier behaviour options, mirroring the types of traffic scenarios assessed in both the KZD questionnaire and WRBTV test. The situations required responses to potential hazards similar to those described in KZD items (e.g., approaching pedestrians near the roadway, encountering stopped vehicles, driving in conditions of reduced visibility) and decision points conceptually analogous to WRBTV scenarios (judging critical distances and appropriate caution levels). This correspondence was intentional, allowing examination of whether drivers’ self-reported tendencies (KZD), laboratory-based risk judgements (WRBTV), and actual simulated driving behaviours (truck simulator) converged in predicting traffic offence history.

Specifically, these situations required drivers to respond to various potential hazards, including

- Intoxicated pedestrians near the roadway (2 situations): These situations presented unpredictable human hazards requiring defensive speed reduction, corresponding to KZD items assessing caution around vulnerable road users.
- Mandatory stop signs (2 situations): These situations assessed regulatory compliance, directly corresponding to KZD items about obeying traffic signs.
- Railroad crossing: This situation required appropriate caution when approaching crossings, mirroring both KZD and WRBTV scenarios involving mandatory stops.
- Pedestrians potentially forcing priority: This situation tested anticipation of pedestrian behaviour, corresponding to hazard perception items in both KZD and WRBTV.
- Trucks emerging from subordinate roads: This situation assessed yielding behaviour and anticipation of other vehicles’ actions.
- Animals (dog and deer) at the roadside (2 situations): These situations required appropriate speed reduction for unpredictable hazards.
- Accident scenes: This situation tested appropriate caution when passing unusual roadway events.
- Reduced visibility conditions due to smoke: This situation assessed speed adaptation to environmental hazards, corresponding to KZD items about driving in adverse conditions and the relevant traffic situations in the WRBTV test.

**Figure 1:**
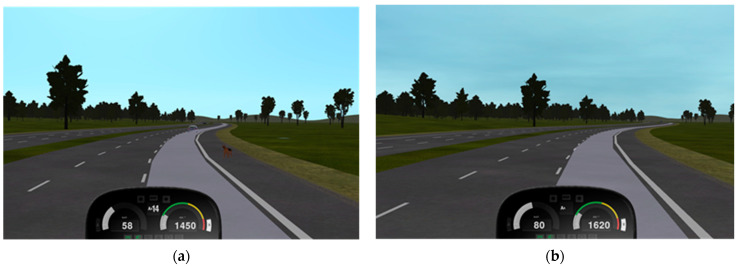
Example decision-making situation from the experimental driving scenario and the corresponding control drive in the truck simulator. *Note:* Image (**a**) shows a truck approaching a potential hazard, i.e., a dog on the right-hand roadside, whereas view (**b**) shows the control drive without any road threat. Drivers’ responses to such situations, particularly speed reduction patterns, were recorded to assess risky driving tendencies. The scenario was conducted under optimal conditions (daylight, dry road surface, good visibility) to capture voluntary safety behaviours rather than environmentally forced responses.

All scenarios were presented under optimal driving conditions (daylight, dry roads, good weather) to ensure drivers’ choices reflected personal tendencies rather than environmental constraints. This design decision was critical, as by eliminating external factors that would force cautious behaviour, the scenarios revealed individual differences in voluntary adoption of safety margins—a key indicator of risk propensity. An example of a decision-making situation from the experimental scenario and the corresponding control drive in the truck simulator is presented in Figure 1.

- Speed metrics: Rationale and validation

The simulator’s data acquisition system recorded multiple driving parameters at a 60 Hz frequency, providing high-resolution temporal data throughout each scenario. For each driving situation, the system captured five fundamental speed-related indicators:

- Initial speed (measured at the defined entry point of each situation);
- Final speed (measured at the defined exit point);
- Minimum speed (lowest recorded speed within the situation);
- Maximum speed (highest recorded speed within the situation);
- Average speed (mean value calculated from all measurements sampled at 60 Hz throughout the situation).

The selection of speed variability as our primary simulator metric was grounded in both theoretical and empirical considerations. From a theoretical perspective, Wilde’s risk homeostasis theory (Wilde, 1982, 1994) predicts that drivers with different subjectively accepted risk levels will exhibit different patterns of speed adjustment when encountering potential hazards. Drivers who maintain larger safety margins should demonstrate greater speed variability, proactively reducing speed when perceiving hazards, whereas drivers with higher risk acceptance should maintain more consistent speeds regardless of situational demands. This theoretical framework suggests that speed variability serves as a behavioural manifestation of individual differences in subjectively acceptable risk levels.

Empirically, previous research has established speed variability as a valid discriminator between accident-involved and accident-free drivers. Af Wåhlberg (2008) demonstrated that vehicle speed changes constitute a key risk indicator, with the magnitude of a driver’s speed changes positively correlating with accident involvement when reflecting appropriate adaptive responses to road hazards. Our own validation work (Baran et al., 2026, manuscript submitted for publication) further demonstrated that this metric successfully differentiated drivers based on traffic violation history. Unlike simple mean speed, which may be influenced primarily by speed limit compliance, speed variability captures dynamic behavioural adaptation to changing risk conditions—a more direct indicator of safety-relevant decision-making processes. This metric reveals whether drivers maintain appropriate safety margins by reducing speed when approaching potential hazards, with greater speed variability in hazardous situations characterising safer drivers who proactively respond to potential risks.

Based on previous validation research (Baran et al., 2026, manuscript submitted for publication), the most diagnostically valuable speed indicator and the most discriminative decision-making situations were identified. This prior study demonstrated that the difference between maximum and minimum speed achieved the highest reliability coefficient (α = 0.71) and showed expected relationships with drivers’ history of traffic violations. The selection of speed variability as a key indicator aligns with findings from other simulator studies (Michaels et al., 2017; Wu et al., 2023), which have consistently identified variations in speed parameters as effective measures for discriminating between safe and risky driving behaviours. Furthermore, previous research (Baran et al., 2026, manuscript submitted for publication) revealed that only 5 of the original 12 situations (specifically, those situations which involved encounters with intoxicated pedestrians, a railroad crossing, a deer by the roadside, an accident scene, and a dog by the roadside) significantly differentiated drivers’ behaviour patterns.

Consequently, for the current analysis, the average of the maximum–minimum speed differences (speed delta) across these five validated decision-making situations was utilised as the primary simulator performance metric. This approach ensured focus on the most reliable and valid indicator of risky driving tendencies, consistent with research demonstrating that speed-based metrics can effectively identify risky driving behaviours in professional driver populations (Baran et al., 2026, manuscript submitted for publication).

#### 2.2.2. Self-Report Questionnaires

The study employed two complementary self-report instruments to capture different aspects of driver characteristics relevant to traffic safety.

- Road Traffic Behaviours Questionnaire (KZD)

The Road Traffic Behaviours Questionnaire (KZD; Baran et al., 2021) consists of 35 statements, including 20 diagnostic items measuring the tendency to take risks on the road and 15 buffer ones. The 20 diagnostic items assess specific risky driving behaviours, including speeding, driving through red lights, forcing right of way, overtaking on continuous lines, driving without seat belts, overtaking in low visibility, driving while fatigued, driving at high speed in poor weather, not reducing speed at railway crossings, changing lanes without signalling, racing with other cars, parking in forbidden places, honking at slow drivers, cutting in on other cars, and texting while driving. The 15 buffer items describe neutral or common driving situations (e.g., shopping at service stations, seeing collisions, using GPS) that do not directly indicate risky behaviour but reduce response bias and disguise the questionnaire’s true purpose. The questionnaire was developed based on driving behaviours identified in the literature as the most frequent causes of road accidents (Baran et al., 2021). Respondents report the frequency of engaging in listed road traffic behaviours on a 4-point Likert scale ranging from 0 (never) to 3 (often). The final score is calculated by summing responses to the 20 diagnostic items, with possible scores ranging from 0 to 60 points, where higher scores indicate a stronger tendency toward risky driving behaviour. The Polish version of the KZD demonstrates good psychometric properties with a Cronbach’s alpha reliability coefficient of 0.88 for the diagnostic items. The complete questionnaire is available in Baran et al. (2021).

- Impulsiveness–Venturesomeness–Empathy Questionnaire (IVE)

The second self-report measure, the IVE Questionnaire (Impulsiveness–Venturesomeness–Empathy), represents a general personality assessment approach. This instrument is a Polish adaptation (Jaworowska, 2011) of Eysenck and Eysenck’s (1978) questionnaire measuring three distinct personality dimensions theoretically linked to risk behaviour across various contexts, including driving. The IVE uses a dichotomous response format whereby respondents answer each item with ‘Yes’ or ‘No’. Each subscale is scored separately by summing the number of items answered in the keyed direction, yielding separate scores for Impulsiveness, Venturesomeness, and Empathy. Higher scores on each subscale indicate higher levels of the respective trait. The Impulsiveness scale assesses tendencies toward unplanned, spur-of-the-moment actions without consideration of potential consequences. The Venturesomeness scale measures conscious risk-taking propensity and sensation-seeking, reflecting a willingness to engage in stimulating but potentially dangerous activities. The Empathy scale evaluates sensitivity to others’ emotional states and capacity for perspective-taking, potentially relevant to prosocial behaviour in traffic environments. The Polish adaptation demonstrates acceptable reliability with Cronbach’s alpha coefficients of 0.78 for Impulsiveness, 0.76 for Venturesomeness, and 0.66 for Empathy (Jaworowska, 2011). Unlike the KZD, the IVE assesses general dispositional traits rather than specific driving behaviours, allowing examination of whether broad personality characteristics predict traffic violations in professional drivers.

#### 2.2.3. Performance-Based Test

- Vienna Risk-Taking Test—Traffic (WRBTV)

The Vienna Risk-Taking Test Traffic (WRBTV) provided a standardised performance-based assessment of risk-taking tendencies in traffic-specific contexts. Developed by Hergovich et al. (2011), this computerised test is based on Wilde’s risk homeostasis theory (Wilde, 1982, 1994). Wilde’s theory posits that individuals maintain subjectively acceptable levels of risk through behavioural adjustments—when encountering an objective risk, drivers compare the current degree of risk with their personal acceptable reference value and adjust behaviour accordingly. While this theory has generated debate in the traffic psychology literature, the core premise has received empirical support in various driving contexts (Hergovich et al., 2011). The WRBTV operationalises this construct by measuring behavioural responses to standardised traffic scenarios rather than relying solely on self-report, which may reduce social desirability bias.

The test presents 24 traffic situations (following one practice item). Each scenario is shown twice to participants. During the first viewing, respondents observe the situation to familiarise themselves with the traffic scenario. During the second viewing, they are required to press a key to indicate the distance from the potential hazard at which the described driving manoeuvre becomes critical or dangerous—that is, the point at which they would no longer perform the manoeuvre. The latency time, measured in hundredths of a second from the start of the scenario until the key press, represents the participant’s willingness to accept risk in that situation. Shorter latencies indicate greater risk acceptance (approaching danger more closely before responding), while longer latencies suggest more cautious behaviour (maintaining greater safety margins). The final score is calculated as the mean latency across all test items. The test demonstrates high internal consistency (Cronbach’s α = 0.92; Hergovich et al., 2011).

**Figure 2:**
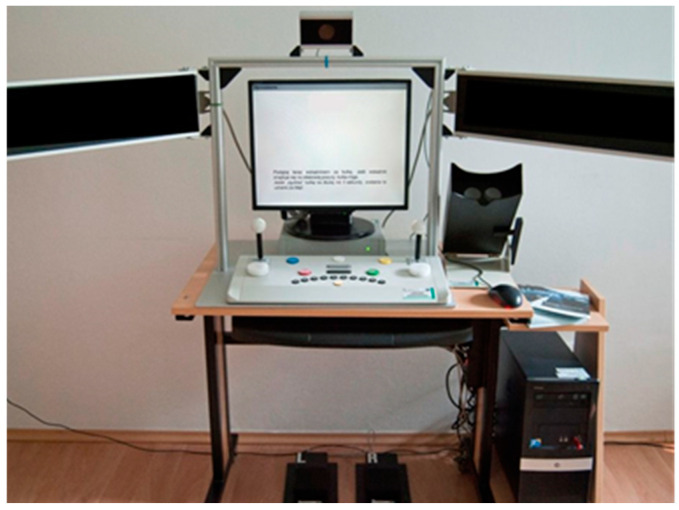
Driver assessment system using the Vienna Risk-Taking Test Traffic (WRBTV).

**Figure 3:**
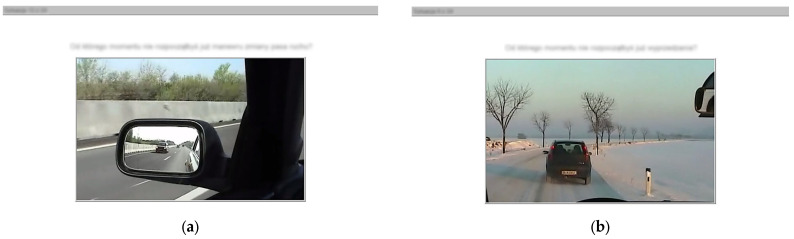
Example traffic situations presented in the WRBTV test (photo source: © SCHUHFRIED GmbH. Used with permission). *Note:* During the test, participants first observe each traffic scenario, then indicate (via key press) the critical distance from the potential hazard at which they would no longer perform the described driving manoeuvre (view (**a**)—“At what point would you not start the changing-lane manoeuvre?” and view (**b**)—“At what point wouldn’t you start overtaking?”). The test measures willingness to accept risk in standardised traffic situations, providing a performance-based complement to self-report measures.

Test administration takes approximately 15 min, including instruction and practice phases. This performance-based approach occupies a middle ground between questionnaires and full simulation, offering standardised assessment of risk decisions without the full immersion of simulator technology. A system for driver assessment with the Vienna Risk-Taking Test Traffic (WRBTV) and examples of traffic situations presented in the test are shown in Figure 2 and Figure 3.

#### 2.2.4. Criterion Variable

Participants’ history of traffic violations served as the criterion variable for assessing the predictive validity of the three measurement approaches. This information was collected through a custom questionnaire covering key aspects of driving history and infractions. Drivers reported specific traffic-related incidents over their entire driving career, including collisions/fender benders, accidents, receiving tickets, police radar stops, licence suspensions (due to accumulated penalty points or excessive speed), and instances of driving under the influence of alcohol. To enhance the accuracy of self-reported violations, the questionnaire employed specific, concrete prompts with defined timeframes and categories, though we acknowledge the inherent limitations of retrospective self-report data discussed in the limitations section. For analytical purposes, participants were classified into two groups based on their self-reported violation history, specifically those with relatively higher numbers of traffic offences and those with relatively lower numbers (see Section 2.3 for cluster analysis methodology). This dichotomisation enabled direct comparison of assessment scores between groups with different real-world driving outcomes, allowing evaluation of each method’s discriminative power. Additionally, participants also reported demographic information like gender, age and education level, driver’s licence categories, and driving experience, i.e., years of professional driving and frequency of driving.

### 2.3. Statistical Analyses

All statistical analyses were conducted using the R statistical package version 4.5.0 with the aid of the psych package version 2.5.3 and the pROC package version 1.18.5.

Descriptive statistics (means, standard deviations, skewness, and kurtosis) were calculated for all measures to evaluate data distribution characteristics. Pearson product–moment correlations were computed to examine bivariate relationships between variables across the entire sample.

To identify groups of participants similar to each other in terms of reported road incidents, cluster analysis was performed on four beforementioned declarative indices (i.e., collisions/fender benders, accidents, received tickets, and police radar stops; licence suspensions and instances of driving under the influence of alcohol were excluded due to zero variance in these variables—none of the participants declared such incidents). A Gower distance matrix was computed to accommodate the mixed ordinal–quantitative nature of the variables. Partitioning Around Medoids (PAM) was then applied to classify 27 participants into two separate clusters (with 16 and 11 subjects). The decision to specify two clusters a priori was based on the research aim to differentiate drivers with higher versus lower traffic offence histories, creating meaningful groups for validating assessment method discriminative power. Group comparisons employed independent samples *t*-tests with Welch’s correction for unequal variances where appropriate. Effect sizes (Cohen’s *d*) with 95% confidence intervals quantified practical significance. Given the exploratory nature and limited sample size, we report both significant results and trends (*p* < 0.10) without applying strict multiple comparison corrections, acknowledging this limitation in interpretation.

Prior to examining assessment method differences, we compared the two identified driver groups on key demographic variables. Independent-samples *t*-tests revealed no significant differences between the lower-offence group (*N* = 16) and higher-offence group (*N* = 11) in age (*M* = 42.38, *SD* = 9.03 vs. *M* = 40.45, *SD* = 5.74; *t*(24.8) = 0.682, *p* = 0.501) or years of professional driving experience (*M* = 14.75, *SD* = 9.90 vs. *M* = 12.73, *SD* = 4.24; *t*(21.77) = 0.726, *p* = 0.476). These non-significant differences confirm that groups differed primarily in traffic offence history rather than demographic characteristics, strengthening the interpretation of subsequent assessment method comparisons.

## 3. Results

Descriptive statistics for the whole sample of drivers, along with the between-variable correlation coefficients (Pearson product–moment correlation), are presented in Table 1.

Notably, the significant positive correlation between WRBTV and KZD (*r* = 0.49, *p* < 0.01) provides initial evidence of convergent validity between performance-based and self-report measures of risky driving tendencies.

Group comparisons revealed differential predictive validity across assessment methods. The KZD demonstrated the strongest effect (Cohen’s *d* = 0.76), with the offence group scoring higher on problematic driving behaviours. Simulator performance also significantly differentiated groups (Cohen’s *d* = 0.68)—critically, in the theoretically predicted direction: drivers with more traffic offences showed smaller speed reductions (*M* = 11.49, *SD* = 3.14) compared to those with fewer offences (*M* = 16.98, *SD* = 10.59), indicating reduced adaptive safety behaviour in response to potential hazards appearing on the road.

Two other measures with moderate effect size also approached statistical significance: WRBTV (*p* = 0.056) and IVE—Empathy (*p* = 0.059). Neither IVE—Impulsiveness (*p* = 0.383) nor IVE—Venturesomeness (*p* = 0.239) showed meaningful group differences. All results are presented in Table 2.

For significant indicators, ROC (Receiver Operator Characteristic) curve analysis (e.g., Robin et al., 2011) was then performed to additionally assess their differentiating ability.

For the speed reduction in simulated driving, 16 controls versus 11 cases showed moderate predictive validity, with the AUC (area under the curve) being 0.64 (95% CI: 0.42; 0.86). This means that in about 64% of cases, the magnitude of the speed reduction could lead to the correct classification of the subjects. The best cutoff point for the score (calculated with the Youden method, e.g., Perkins & Schisterman, 2006) was above 14.8 points, with 0.91 sensitivity (proportion of actual offenders correctly identified) and 0.5 specificity (proportion of non-offenders correctly classified), so almost all cases were correctly classified, but there was also a visible number of false alarms among controls.

For KZD scores, AUC was 0.7 (95% CI: 0.49; 0.9), which means that in about 70% of cases, the results in the KZD questionnaire could lead to the correct classification of the subjects. The best cutoff point for the score was above 18 points, with 0.82 sensitivity and 0.63 specificity.

For WRBTV scores, AUC was 0.68 (95% CI: 0.46; 0.89), which means that in about 68% of cases, the results in the WRBTV test could lead to the correct classification of the subjects. The best cutoff point for the score was above 7.35 points, with 0.91 sensitivity and 0.5 specificity.

For the Empathy scale, AUC was 0.68 (95% CI: 0.46; 0.89), which means that in about 68% of cases, the results in this IVE subscale could lead to the correct classification of the subjects. The best cutoff point for the score was below 11 points, with 0.55 sensitivity and 0.81 specificity (so a large proportion of cases were not correctly classified, but among the designated ones there were relatively few false alarms).

Overall, these findings reveal distinctive patterns among the measurement tools, i.e., while the simulator, KZD, and WRBTV demonstrated high sensitivity but generated more false alarms (not all drivers with risky scores actually committed offences), empathy showed the opposite pattern—missing many cases (some high-empathy individuals also committed violations), but exhibiting high specificity (when empathy was low, it reliably indicated traffic offenders). Notably, these complementary sensitivity–specificity profiles suggest potential value in combining measures within multi-stage assessment protocols, where high-sensitivity tools could serve initial screening functions, while high-specificity measures could reduce false positives in subsequent evaluation stages.

**Figure 4:**
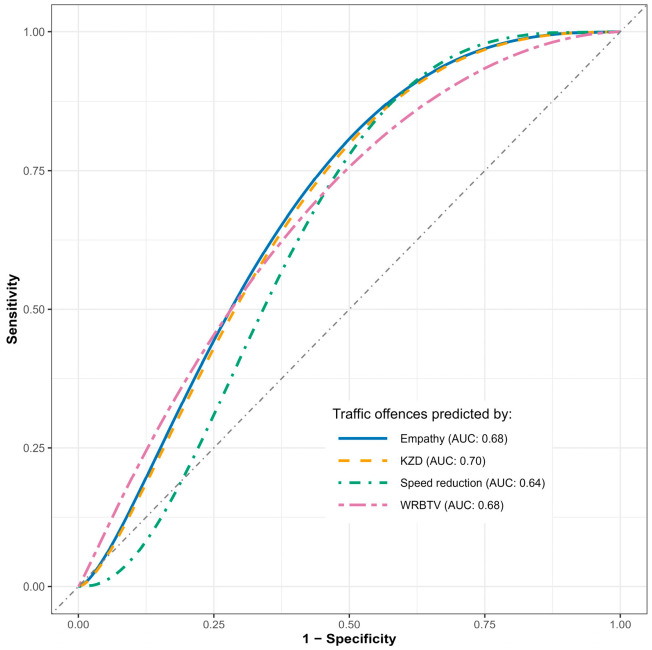
Smoothed ROC curves for predicting the high/low number of traffic offences by different indicators. *Note:* Higher AUC values indicate better discriminative ability, with different measures showing complementary patterns of sensitivity and specificity.

Graphic representation of the ROC curves is presented in Figure 4.

## 4. Discussion

The present pilot study aimed to compare the predictive validity of different assessment methods—specifically self-report questionnaires (KZD, IVE), a computerised performance-based test (WRBTV), and driving simulator performance metrics—for identifying professional drivers, i.e., truck drivers, with traffic offence histories. Specifically, we examined three critical questions: (1) whether domain-specific driving questionnaires outperform general personality measures in identifying at-risk drivers, (2) whether behavioural methods (performance-based tests and simulators) demonstrate superior predictive validity compared to self-report measures, and (3) how these methods compare in their discriminative power when assessed against the same criterion of documented traffic offences. This comparison has direct implications for road safety and public health, as identifying at-risk drivers before they commit violations could prevent accidents and reduce traffic-related morbidity and mortality. Importantly, the findings reveal a clear hierarchy of predictive power, with important implications for both theoretical understanding and practical application in road safety interventions. Overall, our results provided support for H1 (domain-specific KZD outperformed general IVE traits, *d* = 0.76 vs. *d* ≤ 0.30) and H3 (speed variability significantly differentiated groups, *d* = 0.68), while H2 received only partial confirmation (the simulator and WRBTV showed comparable, not superior, predictive validity to KZD), and H4 received support (impulsiveness and venturesomeness showed no significant associations in this professional driver sample, consistent with the hypothesis that these traits may be less predictive in occupationally selected populations than in general driver samples).

First of all, the superior performance of the KZD in discriminating between drivers with and without offences (*d* = 0.76) highlights the value of domain-specific assessment instruments, providing strong support for H1. Our findings regarding the predictive validity of the KZD align with research by Taubman-Ben-Ari et al. (2016), who demonstrated that self-report measures can reliably predict objective driving behaviours. The KZD’s effectiveness may be attributed to its focus on specific, contextualised driving behaviours rather than abstract personality traits, allowing respondents to draw upon concrete behavioural memories when rating item frequency (Baran et al., 2021). This specificity enhances both the ecological relevance of items and the accuracy of self-reports, consistent with the principle that predictors closely matched to criterion behaviours yield stronger associations than general measures (Baran et al., 2021; Martinussen et al., 2017; Taubman-Ben-Ari et al., 2016). Our results parallel those of Martinussen et al. (2017), who found that driver subgroups identified through self-reported driving behaviour questionnaires significantly differed in registered traffic offences, even after controlling for exposure and socio-demographic variables. Similar conclusions were also reached by Wang and Xu (2019) in their naturalistic driving study, which found that self-reported behaviours measured by the Driver Behaviour Questionnaire (DBQ) significantly predicted objective crash risk. Noteworthy, while personality assessments offer broad applicability across different road safety contexts, they appear to sacrifice precision when predicting specific behavioural outcomes like traffic violations. For this reason, the KZD’s focus on driving-related attitudes and self-reported behaviours provides a more direct pathway to understanding real-world driving patterns that may endanger traffic safety and, consequently, public health.

In contrast to the domain-specific KZD, the null findings for general personality traits—impulsiveness and venturesomeness—align with our theoretical predictions (H4) and reflect the distinctive characteristics of professional driver populations. While meta-analytic evidence from Akbari et al. (2019) demonstrates that sensation-seeking—a construct conceptually similar to venturesomeness—positively predicts risky driving behaviours in general driver samples, and Baran et al. (2021) found that impulsiveness significantly predicted risky driving regardless of risk perception among non-professional drivers, our results suggest that such relationships may not generalise to professional drivers operating under distinct occupational constraints. This attenuation of personality–behaviour associations in professional populations likely reflects multiple mechanisms. First, professional driver populations may differ from general samples through selection effects, with extremely impulsive individuals filtered out through licencing requirements, employer screening, or self-selection away from demanding occupational roles. This interpretation aligns with Lucidi et al. (2019), who found that the relationship between personality traits and risky driving behaviours varies significantly across different age groups and driver populations. Second, extensive driving experience may attenuate personality–behaviour relationships through the development of well-practiced safety habits and automatised responses that override impulsive tendencies. This interpretation receives further support from Tseng et al. (2016), who conducted a large-scale study of truck drivers (*N* = 2101) and found that job experience interacted with other factors in complex ways, while demographic factors like age and education and contextual variables such as sleep quality, yearly distance driven, and night driving were the primary predictors of traffic violations. These findings collectively support the broader conclusion from Af Wåhlberg et al. (2017), whose comprehensive meta-analysis concluded that general personality dimensions, i.e., the Big Five personality traits, explain less than 1% of variance in traffic accident records, with effect sizes typically below *r* = 0.1. The observed pattern—whereby domain-specific behavioural measures (KZD) significantly differentiated groups while general personality traits did not—supports the theoretical position that criterion-matched assessments outperform broad personality measures in occupationally selected, experienced driver populations.

Regarding behavioural assessment methods (H2 and H3), both the driving simulator and WRBTV demonstrated meaningful predictive validity, though contrary to H2, they did not outperform the domain-specific self-report measure. Equally noteworthy is the significant predictive validity of simulator-based assessment, specifically the speed variability measure supporting H3. The observed effect size (*d* = −0.68) demonstrates that this behavioural metric—calculated as the average difference between maximum and minimum speeds across five standardised decision-making scenarios—can effectively identify at-risk drivers. This finding aligns with Michaels et al. (2017), who validated simulator-derived measures as indicators of risky driving. However, the direction of the effect—with offence-prone drivers showing smaller speed variability—requires careful interpretation. Counter-intuitively, drivers with more traffic offences demonstrated smaller speed adjustments (*M* = 11.49 km/h) compared to those with fewer offences (*M* = 16.98 km/h). Thus, this pattern suggests that safer drivers proactively modulate speed when approaching potential hazards, creating larger differences between their maximum approach speed and minimum cautionary speed, whereas riskier drivers maintain more constant speeds without substantial defensive reductions. This interpretation aligns with Af Wåhlberg (2008), who argues that vehicle speed changes constitute a key risk indicator, with the sum of a driver’s speed changes positively correlating with their accident involvement when reflecting appropriate adaptive responses to road hazards. Moreover, the significant predictive validity of our simulator-based metrics aligns with findings from Ka et al. (2020), who demonstrated that surrogate safety measures—comparable to our speed variability metric—implemented in driving simulators can effectively identify risky driving behaviours, particularly among commercial drivers.

Beyond the simulator, the marginal significance of the WRBTV (*p* = 0.056) provides convergent evidence for the value of performance-based assessment, though the computerised format appears somewhat less sensitive than full simulation. The moderate correlation between WRBTV and KZD (*r* = 0.49, *p* < 0.01) suggests these methods capture related but partially distinct aspects of risk propensity, with WRBTV assessing perceptual–cognitive risk judgements and KZD measuring self-reported behavioural tendencies. Indeed, this pattern indicates that ecological validity may enhance predictive accuracy, with more realistic assessment contexts yielding stronger criterion relationships relevant to actual traffic safety outcomes.

Overall, the ROC analyses provide additional insights into the practical utility of these measures and address our third research question regarding the potential for combining assessment methods. The moderate AUC values (0.64–0.70) across significant predictors suggest reasonable discriminative ability, though none achieved excellent classification. Notably, the differential patterns of sensitivity and specificity across measures have important practical implications, i.e., high-sensitivity tools (simulator, KZD, WRBTV) may be optimal for initial screening where identifying all potential offenders is crucial, while the high-specificity empathy measure could serve as a confirmatory assessment to reduce false positives. This complementary pattern suggests potential value in multi-stage assessment protocols for professional drivers.

Regarding the practical significance of our findings, the obtained effect sizes (*d* = 0.68–0.76 for significant predictors) represent medium-to-large effects, suggesting meaningful differences between driver groups. The ROC analyses provide further insight into practical utility: AUC values of 0.64–0.70 indicate that these measures can correctly classify drivers at rates substantially better than chance (50%). While these classification rates are moderate rather than excellent, they are consistent with the complex, multifactorial nature of traffic safety, where single measures rarely achieve perfect prediction due to the numerous interacting factors that influence driving outcomes. From a practical screening perspective, these measures could identify approximately 70% of at-risk drivers when used appropriately. This represents a meaningful improvement over current practices in many organisations, which often rely solely on periodic driving record review or no systematic assessment at all.

Furthermore, the relatively modest predictive validity observed across all measures, even for our strongest predictor (KZD, *d* = 0.76), is consistent with broader patterns in traffic safety research. Barraclough et al.’s (2016) meta-analysis found an average correlation of only *r* = 0.18 between traffic offences and crashes, suggesting that the relationship between driving-related variables may be inherently limited by the multifactorial nature of traffic incidents. This underscores the value of our multi-method approach, as relying on any single assessment method would likely miss important variance in driver safety outcomes.

Finally, beyond the hypothesised predictors, an intriguing exploratory finding concerns the protective role of empathy. This variable emerged as a meaningful discriminator in our ROC analyses (AUC = 0.68), with drivers without offence histories showing higher empathy scores (*p* = 0.059), suggesting that interpersonal sensitivity may constitute an overlooked protective factor in traffic safety. This pattern aligns with findings by Baran et al. (2021), who identified empathy as a significant negative predictor of conscious risk-taking in road traffic situations. Experimental and correlational evidence further supports this relationship: Crundall and van Loon (2023) demonstrated that empathy-focused interventions resulted in safer driving behaviours, while Šeibokaitė et al. (2017) found that difficulties in emotion regulation were associated with driving errors and violations. Theoretically, empathetic individuals may better anticipate other road users’ needs and view traffic rules as prosocial obligations rather than mere restrictions—consistent with Vaa’s (2014) framework emphasising emotional processes in driving decisions. This protective role of empathy may be further enhanced by broader value systems, as Mamcarz (2023) demonstrated that adherence to core beliefs strengthens empathy’s influence on driving safety, while Awad-Yassin and Taubman-Ben-Ari (2025) found that sense of community was associated with less maladaptive driving styles.

### Limitations and Future Directions

Regardless of the above, the results obtained in the study should be interpreted within the study’s limitations. The pilot nature of this study, with its small sample size (*N* = 27), constitutes the primary limitation, though the emergence of statistically significant results with moderate-to-large effect sizes (*d* = 0.68–0.76) despite this constraint suggests robust underlying relationships. The sample size restricts statistical power and precludes multivariate analyses that could identify optimal predictor combinations. However, the null findings for impulsiveness and venturesomeness align with our theoretical predictions and prior meta-analytic evidence (Af Wåhlberg et al., 2017), suggesting these results reflect genuine characteristics of professional driver populations rather than insufficient power.

Moreover, our reliance on self-reported offence history introduces potential bias, though the observed group differences suggest meaningful criterion variance remained. As noted by Barraclough et al. (2016), self-reported traffic data typically yield inflated associations compared to archival records, though our use of objective simulator measures helps mitigate this bias. Furthermore, the cross-sectional design limits causal inferences. Thus, longitudinal studies tracking drivers from assessment through subsequent traffic safety records would strengthen conclusions about the public health impact of early identification and intervention. Such designs, as Beanland et al. (2013) suggest, would also reveal whether assessment results remain stable over time or require periodic updating.

In addition, an important consideration for future research concerns the distinction between predicting traffic offences and predicting crashes. Martinussen et al. (2017) found that while driver behaviour questionnaires successfully predicted traffic offences, they failed to differentiate groups regarding crash involvement. This pattern may reflect the complex, multi-causal nature of crashes, where factors beyond individual behaviour, i.e., including other drivers’ actions and environmental conditions, play significant roles.

A further limitation concerns the ecological validity of simulator assessment. While our high-fidelity simulator with a motion platform enhances realism compared to fixed-base systems, participants’ awareness of the simulated environment and absence of real consequences may have influenced behaviour. Hence, some predicted behaviours, like, for example, horn use, were notably absent or under-represented, possibly because drivers recognised that virtual objects would not respond to auditory signals and that risky choices carried no actual penalties. Nevertheless, this awareness effect represents an inherent constraint of simulator methodology that future research should acknowledge when interpreting findings. At the same time, the significant group differences obtained using simulator metrics demonstrate that meaningful behavioural variance can be captured even within these constraints, supporting the utility of simulation-based assessment for professional driver evaluation. The correspondence between our simulator findings and real-world traffic offence histories suggests that the simulator successfully elicited behaviour patterns reflective of individuals’ actual risk-taking tendencies. Independently, future research employing naturalistic driving methodologies could complement simulator findings by validating these patterns in real-world contexts where all consequences of driving decisions are present.

Despite these limitations, the present pilot study provides valuable preliminary evidence for the comparative validity of different driver assessment methods and establishes a foundation for larger-scale investigations. The convergent findings across multiple assessment modalities—with domain-specific questionnaires and simulator metrics both differentiating driver groups—strengthen confidence in the practical applicability of these methods for professional driver screening and offer actionable insights for traffic safety enhancement. The preliminary findings from this pilot investigation suggest potential value in prioritising domain-specific instruments like the KZD over general personality measures when assessing professional drivers and indicate that simulator-based assessment may offer complementary information for safety-critical positions. However, these recommendations should be considered tentative pending replication with larger samples and validation against prospective safety outcomes rather than retrospective offence histories. Organisations considering implementation of such assessment protocols should view these tools as components of comprehensive evaluation approaches rather than standalone decision-making instruments. Furthermore, the potential protective role of empathy warrants attention, as demonstrated by Crundall and van Loon (2023), who showed that empathy-focused interventions successfully modified drivers’ attitudes toward vulnerable road users.

Building upon these practical implications, several directions for future research also emerge. Primarily, future studies should validate simulator findings through naturalistic driving research, such as that conducted by Zhu et al. (2025), which examines how personality factors translate into actual on-road behaviours. Moreover, investigations should consider drivers’ risk perception as a potential moderating variable, as some personality traits may primarily influence behaviour among drivers who consciously recognise road risks (Baran et al., 2021). The spectrum of psychological factors could also be expanded to include novel constructs such as “spiritual fitness”, which Mamcarz (2023) has proposed as a potential mediator between personality traits and risky driving behaviours. Furthermore, future research might also benefit from incorporating alternative domain-specific measures such as the Dula Dangerous Driving Index (DDDI; Dula & Ballard, 2003), which includes subscales assessing risky and aggressive driving behaviours, to further validate the relative predictive utility of different self-report instruments in professional driver populations. Additionally, given cultural variations in personality-driving relationships, as shown by Bhosale and Reddy (2019) in India, cross-cultural studies are needed to inform globally applicable road safety interventions. Finally, replication with larger samples and objective offence records would further validate the promising patterns observed in this pilot investigation.

## 5. Conclusions

This pilot study provides preliminary comparative evidence regarding the predictive validity of multiple driver assessment methods—an understudied area with substantial public health implications. Domain-specific questionnaires and simulator-based metrics showed promising ability to differentiate professional drivers with and without traffic offence histories, whereas general personality traits showed limited associations, consistent with theoretical predictions regarding occupational selection effects. However, given the pilot nature of this investigation, the limited sample size, and the reliance on self-reported criterion data, these findings must be interpreted cautiously. Future research with prospective designs, objective safety records from company or regulatory databases, and substantially larger samples would be valuable to determine whether the patterns observed here generalise to operational contexts and predict future safety outcomes rather than historical offence patterns. Nevertheless, this study establishes a methodological foundation for larger-scale investigations and suggests that evidence-based screening protocols combining domain-specific questionnaires with behavioural assessment may contribute to proactive traffic safety interventions.

## Figures and Tables

**Table 1:** Descriptive statistics and the correlation coefficients for the whole sample of truck drivers (*N* = 27).

Assessment Method	*M*	*SD*	Skewness	Kurtosis	Pearson’s *r*				
					[2]	[3]	[4]	[5]	[6]
[1] Speed reduction in simulated driving	14.74	8.72	1.59	2.44	−0.29	−0.30	0.01	0.06	−0.05
[2] WRBTV	7.99	1.51	−0.06	−0.27		0.49 *	0.28	0.02	0.11
[3] KZD	18.67	7.40	−0.01	−0.45			0.33	−0.10	0.28
[4] IVE—Impulsiveness	3.85	3.43	0.46	−1.01				0.10	0.24
[5] IVE—Empathy	11.11	3.13	−0.45	−0.89					−0.31
[6] IVE—Venturesomeness	7.93	3.60	0.10	−0.30					

* *p* < 0.01.

**Table 2:** Comparison of assessment methods in differentiating drivers with lower (Gr. 1, *N* = 16) and higher (Gr. 2, *N* = 11) numbers of traffic offences.

Assessment Method	Gr. 1 *M* (*SD*)	Gr. 2 *M* (*SD*)	*t*	*df*	*p*	Cohen’s *d*	95% CI for Cohen’s *d*
Speed reduction in simulated driving	16.98 (10.59)	11.49 (3.14)	1.95	18.63	0.033 *	0.68	[−0.12, 1.46]
WRBTV	7.61 (1.53)	8.54 (1.37)	−1.65	23.21	0.056 †	0.66	[−0.14, 1.44]
KZD	16.56 (7.43)	21.73 (6.50)	−1.91	23.43	0.034 *	0.76	[−0.04, 1.55]
IVE—Impulsiveness	3.69 (3.65)	4.09 (3.24)	−0.30	23.25	0.383	0.12	[−0.65, 0.89]
IVE—Empathy	11.88 (3.24)	10.00 (2.72)	1.63	23.89	0.059 †	0.64	[−0.15, 1.42]
IVE—Venturesomeness	7.50 (3.52)	8.55 (3.80)	−0.72	20.51	0.239	0.30	[−0.48, 1.07]

* *p* < 0.05; † 0.05 < *p* < 0.10 (marginally significant). *Note:* Negative *t*-test values indicate higher scores in the group with a higher number of traffic offences; positive values indicate higher scores in the group with a lower number of traffic offences.

## Data Availability

The data presented in this study will be made available by the corresponding author upon reasonable request.
